# Persistence of IgE-Associated Allergy and Allergen-Specific IgE despite CD4+ T Cell Loss in AIDS

**DOI:** 10.1371/journal.pone.0097893

**Published:** 2014-06-04

**Authors:** Katharina Marth, Eva Wollmann, Daniela Gallerano, Portia Ndlovu, Ian Makupe, Rudolf Valenta, Elopy Sibanda

**Affiliations:** 1 Division of Immunopathology, Department of Pathophysiology and Allergy Research, Center of Pathophysiology, Infectiology and Immunology, Medical University of Vienna, Vienna, Austria; 2 Christian Doppler Laboratory for Allergy Research, Division of Immunopathology, Department of Pathophysiology and Allergy Research, Center of Pathophysiology, Infectiology and Immunology, Medical University of Vienna, Vienna, Austria; 3 Asthma, Allergy and Immune Dysfunction Clinic, Harare, Zimbabwe; King’s College London, United Kingdom

## Abstract

The infection of CD4+ cells by HIV leads to the progressive destruction of CD4+ T lymphocytes and, after a severe reduction of CD4+ cells, to AIDS. The aim of the study was to investigate whether HIV-infected patients with CD4 cell counts <200 cells/µl can suffer from symptoms of IgE-mediated allergy, produce allergen-specific IgE antibody responses and show boosts of allergen-specific IgE production. HIV-infected patients with CD4 counts ≤200 cells/µl suffering from AIDS and from IgE-mediated allergy were studied. Allergy was diagnosed according to case history, physical examination, skin prick testing (SPT), and serological analyses including allergen microarrays. HIV infection was confirmed serologically and the disease was staged clinically. The predominant allergic symptoms in the studied patients were acute allergic rhinitis (73%) followed by asthma (27%) due to IgE-mediated mast cell activation whereas no late phase allergic symptoms such as atopic dermatitis, a mainly T cell-mediated skin manifestation, were found in patients suffering from AIDS. According to IgE serology allergies to house dust mites and grass pollen were most common besides IgE sensitizations to various food allergens. Interestingly, pollen allergen-specific IgE antibody levels in the patients with AIDS and in additional ten IgE-sensitized patients with HIV infections and low CD4 counts appeared to be boosted by seasonal allergen exposure and were not associated with CD4 counts. Our results indicate that secondary allergen-specific IgE production and IgE-mediated allergic inflammation do not require a fully functional CD4+ T lymphocyte repertoire.

## Introduction

IgE-associated allergy is a frequent problem in central Africa [1]–[4]. House dust mites, grass pollen and various foods are important allergen sources in Africa as it has been demonstrated by skin testing and IgE serology [5], [6]. Infectious diseases and parasite infestations are frequent in central Africa and it has been demonstrated that schistosome infestations are negatively associated with allergic diseases [7]. However, according to clinical observations allergy is quite common in Africa and does not seem to follow strictly the rules of the Hygiene hypothesis [8].

Besides parasitic infestations HIV infections represent one of the major health problems in central and southern Africa. The prevalence of HIV infections in Zimbabwe exceeds 15% (UNAIDS global report 2010; http://www.unaids.org/globalreport/) of the population. HIV infections profoundly affect the immune system leading to a severe loss of functional CD4+ T lymphocytes. These changes may cause alterations of the Th1/Th2 cytokine balance, polyclonal hypergammaglobulinaemia and increases in total serum IgE levels [9]. Since CD4+ T cells producing IL-4 are essential for the class-switch towards IgE and the development of IgE-associated allergy we were interested to investigate the effects of severe CD4+ T cell loss on allergic symptoms and allergen-specific IgE production. Therefore, we have analyzed allergic symptoms and allergen-specific IgE production in HIV-infected patients with <200 CD4+ cells/µl and in HIV-infected patients with low CD4 counts in the range of 200–700 cells/µl.

## Methods

### Patients’ Sera

Sera were obtained from patients of the Allergy and Immune Dysfunction out-patients clinic in Harare, Zimbabwe. Patients were either referred for treatment of allergy and, on subsequent investigation were found to be HIV positive or were referred for the management of HIV/AIDS and were found to suffer from allergic diseases. HIV-infected patients with low CD4+ T cell counts and CD4+ T cell counts below 200/µl were identified who also suffered from IgE-mediated allergies (Tables 1 and 2; Tables S2 and S3). The range of CD4 counts for healthy individuals according to WHO definitions is 500–1500 cells/µl.

**Table 1:** Demographic, clinical and immunological characterization of eleven HIV-infected allergic patients suffering from AIDS according to the CDC classification.

Patient#	Age	Sex	CD4 counts at sampling	Allergy Symptoms	Allergens tested positive in SPT, MAST CLA or Euroline IgE assay
**1**	40	F	115	RC	Cat, Dog, Horse, Guinea pig, Hamster, Rabbit, Mugwort, Parietaria, Ragweed, Olive tree, Birch, Juniper, Grass mix, Pine mix, Hazelnut, Peanut, Walnut, Almond, Egg, Casein, Potato, Celery, Codfish, Shrimp, Apple, Wheat flour, Sesame, Soy bean, Peach, Latex, Penicillium, Cladosporium, Aspergillus, Alternaria, Cockroach, Der p
**2**	36	M	115	R	Rabbit, Mugwort, Parietaria, Ragweed, Olive tree, Birch, Juniper, Grass mix, Pine mix, Hazelnut, Peanut, Walnut, Almond, Egg, Casein, Potato, Celery, Codfish, Shrimp, Apple, Wheat flour, Sesame, Soy bean, Peach, Latex, Penicillium, Cladosporium, Aspergillus, Alternaria, Cockroach, maize pollen, Feathers, Soy
**3**	43	M	150	R	Der p, Cotrimoxazole
**4**	58	F	200	R	Der p
**5**	37	F	77	AB	Wheat flour, Rice, Soy bean, Peanut, Haselnut, Carrot, Potato, Apple, Grass mix, Birch tree, Mugwort, Der p, Der f, Dog epithelia
**6**	30	F	135	R	Cat, Dog, Ragweed, Birch, Juniper, Pine mix, Peanut, Walnut, Almond, Egg, Casein, Potato, Celery, Codfish, Shrimp, Apple, Wheat flour, Soy bean, Peach, Latex, Penicillium, Cladosporium, Aspergillus, Alternaria, Cockroach, Der p
**7**	40	F	142	R, AB	n.d.
**8**	39	M	171 (120–217)	R	Der p, Grasses
**9**	47	F	108	AB	5 Grasses Mix, Horse, Der p, Der f, Dog epithelia
**10**	67	F	117 (117–142)	R	Meat, Carp, Horse epithelia, Hens egg yolk, Goat epithelia, Hen feather, Reed, Grasses, Cereals, Der p, Der f, Storage mite
**11**	25	F	184 (4–184)	U, Angioedema	Mutton, Hens egg Yolk, Grasses, HDM

Displayed are age, sex, CD4 T cell counts at date of serological IgE diagnosis or range of CD4 counts for follow-up sera. Allergic symptoms and positive results obtained by skin prick testing and determination of allergen-specific IgE are summarized.

Abbreviations: F: female; M: male; R: rhinitis; RC: rhinoconjunctivitis; U: urticaria; AB: asthma bronchiale; AP: allergic pharyngitis; SPT: skin prick test; Der p: *Dermatophagoides pteronyssinus*; n.k.: not known;

**Table 2:** Demographic, clinical and immunological characterization of ten HIV infected patients with low CD4 counts.

Patient#	Age	Sex	Range of CD4 counts	AllergySymptoms	Allergens tested positive in SPT, MAST CLA, Euroline IgE assay or allergen sources in ISAC
**12**	49	M	168–205	RC	Alpha-lactalbumin, Der p, Dog epithelia, Cypress, Cedar,
**13**	32	F	62–106	R, AP	Cedar, Bermuda grass, Cypress, Timothy grass
**14**	50	F	110–677	AB	Der p, Der f, 4 grains mix, Bermuda grass, Cypress
**15**	37	M	375–518	R	Der p, Der f, Dog epithelia, cat, peanut, dog epithelia, Bermuda grass, Plane, Wesp, Almond, Peach
**16**	43	M	244–288	R, AP	Cypress, Cedar
**17**	26	F	261–741	R	Glutein, peanut, Altanaria, Cypress, Cedar, Der f, Der p, Walnut, Timothy grass, Bermuda grass, Plane
**18**	39	F	209–223	R	Der f, Der p
**19**	50	M	257–415	R	Der p, Der f, Meat, Aspergillus
**20**	40	F	488–722	n.k.	Der p, Der f, Dog epithelia, Alternaria, Cypress, Bermuda grass, Cedar, Olive, Plane, Timothy grass
**21**	47	M	486–501	R	Cypress, Cedar, Der f, Der p, Latex

Displayed are age, sex and ranges of CD4 counts for follow-up sera. Allergic symptoms and positive results obtained by skin prick testing and determination of allergen-specific IgE are summarized.

Abbreviations: F: female; M: male; R: rhinitis; RC: rhinoconjunctivitis; U: urticaria; AB: asthma bronchiale; AP: allergic pharyngitis; SPT: skin prick test; Der p: *Dermatophagoides pteronyssinus*; n.k.: not known;

### Ethical Considerations

The study was approved by the Institutional Ethics Committee, and informed consent was obtained from the subjects. Anonymized sera were analyzed for allergen-specific IgE antibodies with approval by the ethics committee of the Medical University of Vienna, Austria.

### HIV Status and Disease Staging

The HIV-positive serological status was diagnosed with Determine HIV1/2 (Abbott Diagnostic Division, Hoofddorp, The Netherlands) and Capillus HIV-1/HIV-2 (Trinity Biotech, Jamestown, NY, US) reactive rapid test assays. CD4+ T lymphocyte numbers were determined using a Becton Dickinson FACSCan flow cytometer and Tritest reagents (Becton Dickinson) reagents. The combination of the clinical and laboratory information allowed the patients to be stratified according to the CDC classification system (1993 and 2008) [10], [11].

### Allergy Diagnosis

The clinical diagnosis of allergy included a structured personal history of allergy, a clinical examination of target organs and correlation of symptoms with allergen exposure. Skin prick tests (SPT) were performed by using extracts from a standard panel of inhaled and ingested allergen source extracts according to case history (Stallergenes, Anthony, France) (Tables S1, S2, S3). The positive and negative controls were 10 mg/ml histamine hydrochloride and phenolated glycerol-saline, respectively. Skin test wheal reactions were measured after 15 minutes and considered positive if a patient had a mean wheal diameter of ≥3 mm. Patients were checked for late phase allergic symptoms such as atopic dermatitis by inspection and/or atopy patch testing (i.e., epicutaneous allergen administration followed by inspection after 48 hours for symptoms of eczema).

Allergen-specific IgE antibodies were determined with the CLA assay (Hitachi Chemical Diagnostics, Mountain View, CA, US) or the EUROLINE IgE assay (EUROIMMUN AG, Lübeck, Germany) (Table S1). For 6 patients (Table 1: patients #5, 7, 8, 9, 10, 11) the molecular allergen profile was established with a customized allergen microarray containing purified recombinant and natural allergens (Thermo Fisher, Uppsala, Sweden) (Note S1) [12].

**Figure 1:**
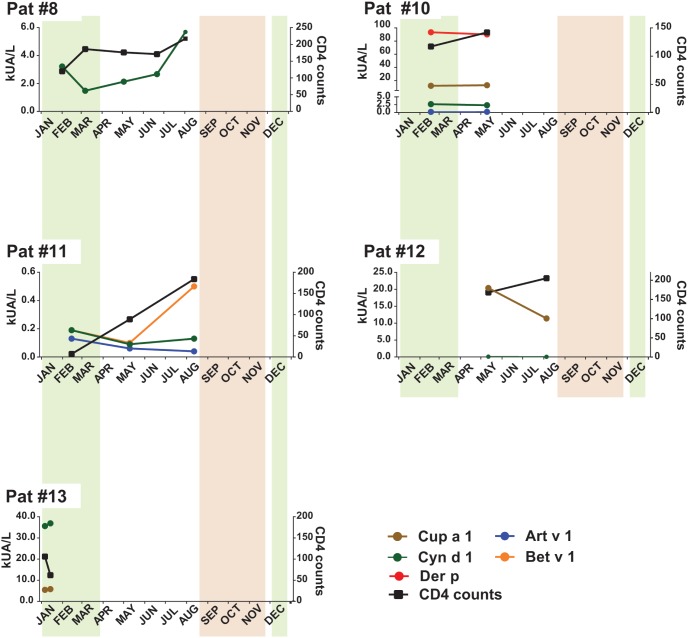
Allergen-specific IgE antibody levels (kUA/L) and CD4 counts in HIV-positive patients with AIDS (#8–11) and in HIV-positive patients with CD4 counts below 200/µl (#12–13). CD4 counts (right y-axes) and allergen-specific IgE levels (left y-axes) measured in serum samples obtained at different points of time (x-axes) are shown. The usual periods of the grass and tree pollen season is indicated in green and brown, respectively.

**Figure 2:**
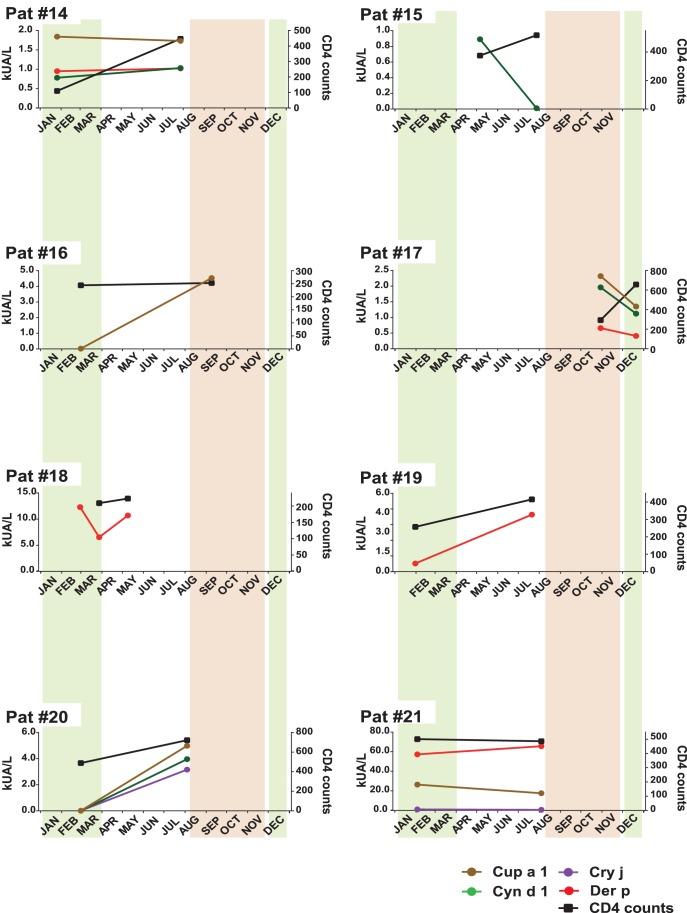
Allergen-specific IgE antibody levels (kUA/L) and CD4 counts in HIV-positive patients with low CD4 counts (#14–21) for whom sequential serum samples were available. CD4 counts (right y-axes) and allergen-specific IgE levels (left y-axes) measured in serum samples obtained at different points of time (x-axes) are shown. The usual periods of the grass and tree pollen season is indicated in green and brown, respectively.

Sera from additional HIV-infected patients (Table 2, Table S3) containing allergen-specific IgE with CD4 counts <200 cells/µl (i.e., patients #12, 13; Fig. 1) and between 200–700 cells/µl (i.e., patients #14–21; Fig. 2) were analyzed for allergen-specific IgE antibodies by micro-array and then studied regarding changes of allergen-specific IgE in follow up serum samples. Allergen-specific IgE levels in the follow up serum samples were quantified by ImmunoCAP (Thermo Fisher) measurements.

## Results

### Patients with AIDS Suffer from IgE-mediated Allergic Symptoms and Produce Allergen-specific IgE

The mean CD4 counts in patients with AIDS were 150 cells/µl (ranging from 77 to 200 cells/µl) (Table 1, Table S2) and each of the studied patients fulfilled the criteria of AIDS according to the CDC [10], [11]. Interestingly, all patients had typical symptoms of IgE-mediated allergies which most likely were due to IgE-mediated mast cell activation. Accordingly, they also showed positive skin prick test reactions indicating allergen-induced mast cell activation by IgE-allergen immune complexes (Table S2). In fact, most (i.e., 73%) subjects suffered from acute allergic rhinitis, 27% from acute allergic bronchial asthma, one from conjunctivitis and one from urticaria and angioedema (Table 1, Table S2). Notably, none of the patients exhibited late phase symptoms such as atopic dermatitis or other T cell-mediated allergic symptoms. There were no differences regarding the other symptoms of allergic disease between HIV positive patients at any stage of their disease and HIV-negative allergic patients seen in the outpatient clinic (data not shown).

Sensitizations to 49 different allergen sources were found in the patients either by SPT and/or serological IgE testing (i.e. CLA assay, EUROIMMUN assay, ImmunoCAP ISAC). House dust mite (HDM) allergens were recognized by 81% of the patients while 63% reacted with grass pollen. Sensitizations to apple, birch, codfish, dog dander, egg, peanut, potato, soy bean and wheat flour were diagnosed in 4/11 patients (Table 1 and Tables S2). Sensitization to birch pollen may reflect sensitisation to cross-reacting allergens in pollen of local trees and plant food containing PR10 protein allergens.

The allergen micro-array profile analyzed in approximately half of the patients also confirmed that house dust mite allergens are the most frequent allergen components (data not shown). These results are in agreement with earlier studies [4], [6] where sensitizations to HDM, grass pollen and foods were most frequently found in allergic patients from central and southern Africa.

### Allergen-specific IgE Levels of Allergic Patients with AIDS Mainly Follow Allergen Exposure and are not Associated with CD4 Counts

For three AIDS patients (i.e. #8, #10, and #11; Table 1, Table S2) follow-up sera, collected between February and August 2011 were available. Interestingly, the levels of grass pollen allergen (Cyn d 1) specific IgE seemed to depend on grass pollen exposure because they increased after grass pollen exposure around March but did not follow strictly the CD4 cell counts (Fig. 1). These results are in agreement with data from other studies showing that respiratory allergen exposure boosts allergen-specific IgE production [13]. The monitoring of allergen-specific IgE levels in patient #10 (Fig. 1) showed that this patient exhibited highly elevated house dust mite allergen-specific IgE levels despite low CD4 cell counts. Allergen-specific IgE levels in patient #11 also did not follow CD4 cell counts. While CD4 cell counts increased, IgE levels to Art v 1 and Cyn d 1 declined and increased for Bet v 1 (Fig. 1). Also in two additional IgE sensitized patients i.e., patient #12 and patients #13 (Table 2, Table S3) with CD4 counts below 200 cells/µl, allergen-specific IgE levels did not follow CD4 counts. In patient #12, IgE-specific for the tree pollen allergen Cup a 1 decreased outside the pollen season, while CD4 counts increased (Fig. 1). In patient #13, IgE specific for the grass pollen allergen Cyn d 1 and for the tree pollen allergen Cup a 1 went up in the pollen season while CD4 counts decreased (Fig. 1). When we analyzed eight additional HIV-infected patients (Table 2, Table S3) with reduced CD4 counts (i.e., 200–700/µl) similar observations were made (Fig. 2). Patients with low CD counts showed increases of allergen-specific IgE (e.g., patients #16, 18, 20), in other patients allergen-specific IgE stayed unaltered or dropped, despite increases of CD4 counts (e.g., patients #14, 15, 17). Only in two of the eight patients studied in Fig. 2 (i.e., patients #19 and #20), allergen-specific IgE levels and CD4 counts showed a similar behaviour (Fig. 2).

## Discussion

It is well established that T cell help in the form of CD4 cells producing Th2 cytokines is necessary for the induction of class switch towards IgE production in allergic patients during primary sensitization [14]. Regarding the secondary IgE production in already sensitized allergic patients less is known. In fact, it has been shown that adult allergic patients do not acquire sensitizations against new allergens but exhibit a constant IgE reactivity profile against a defined set of allergens [13], [15]. The levels of allergen-specific IgE in already sensitized allergic patients decline in the absence of allergen exposure and increase upon allergen exposure [13]. Interestingly, increases of allergen-specific IgE levels can be induced by isolated allergen contact via the nasal mucosa [13] and it may be speculated that either local IgE production in the nasal mucosa or stimulation of the adjacent lymphatic tissues is responsible for this event [16]. Interestingly, the boost of secondary allergen-specific IgE production can only be induced with allergen molecules containing the intact IgE epitopes but not with allergen-derivatives containing only T cell epitopes [17]. Using a murine model of primary allergic sensitization and established secondary IgE responses towards a major grass pollen allergen and co-stimulation blockade, it was found that only primary allergic sensitization but not secondary allergen-specific IgE production was dependent on T cell help [18]. The latter findings thus indicated that the secondary allergen-specific IgE production does not necessarily require T cell help. Since HIV infection leads to a damage of CD4+ T cells, we thought to investigate whether HIV-infected patients with AIDS and severely reduced CD4 T cell counts continued to suffer from IgE-associated allergic symptoms and to produce allergen-specific IgE.

Our results showed that patients with severely reduced CD4 cell counts continued to suffer from symptoms of IgE-mediated allergy and produced allergen-specific IgE antibodies. More importantly, we found that certain allergic patients with an HIV infection still showed increases of allergen-specific IgE production despite low CD4 cell counts. The lack of late phase allergic symptoms due to allergen-specific T cells (e.g., atopic dermatitis) in the studied patients indeed indicates that the activity of allergen-specific CD4 cells was compromised.

Two reports, one showing that immune complexes regulate the fate of germinal center B cells, in particular their affinity maturation [19], and another showing that B cells see antigen on follicular dendritic cells [20] suggest possible mechanisms for B cell activation without strong engagement of T cell help. In these scenarios one could imagine that memory B cells experience cross-linking of membrane immunoglobulin either by immune complexes or antigen on follicular dendritic cells and then respond by transformation into plasma cells and/or antibody production even in the absence of T cell help.

Further experiments will be required to test this hypothesis but our results are in agreement with data obtained by other groups investigating the possible associations in the changes of allergen-specific IgE levels with changes in total IgE levels in HIV-infected patients with AIDS. Also these studies observed that patients with AIDS and low CD4 cell counts mounted allergen-specific IgE responses in serum but they have not considered interpreting these findings regarding T cell dependence of IgE production [21], [22]. Although it has been found that HIV can favour a shift to the TH0 phenotype and preferentially replicates in CD4 T cells producing TH2-type cytokines [23] it does not lead to a switch from the Th1 to the Th2 cytokine phenotype [24]. It is therefore unlikely that the persistence of allergy and allergen-specific IgE production is due to a selective loss of Th1 cells. We could not investigate the effects of allergen exposure on freshly isolated T cells and their cytokines from the patients in Africa due to the lack of tissue culture facilities there but it is conceivable that in patients with low CD4 cell counts also the production of cytokines upon antigen stimulation will be impaired.

In summary, our study indicates that allergic patients suffering from AIDS and severely reduced CD4 cells continue to produce allergen-specific IgE antibodies which can be boosted by allergen exposure and do not follow CD4 cell counts. These patients suffer from IgE-mediated acute allergic symptoms. CD4 T lymphocytes were not totally absent in our patients but their numbers were severely reduced and accordingly no delayed type hypersensitivity reactions were observed in the patients. Together with data from a murine model of IgE-mediated allergy performed under co-stimulation blockade our results support the possibility that the secondary IgE response in allergy does not require a fully functional T cell repertoire. This finding is unexpected but may be important for the development of therapeutic strategies for IgE-associated allergies.

## Supporting Information

Table S1 **Allergen extracts tested.** Panel of allergen extracts for diagnosis by skin prick testing and IgE serology (CLA assay, Euroline). Abbreviations: Der p: *Dermatophagoides pteronyssinus.*
(DOC)

Table S2 **Demographic, clinical and immunological characterization of eleven HIV-infected allergic patients suffering from AIDS according to the CDC classification.** Displayed are age, sex, HIV status, year and CD4 counts when AIDS was diagnosed, CD4 counts at the date of IgE serology and range of CD4 counts for follow-up sera, viral load, antiretroviral therapy, allergic symptoms, year when allergy was diagnosed, positive allergy diagnosis results obtained by skin prick test and IgE serology by CLA assay or Euroline IgE assay. Abbreviations: F: female; M: male; R: rhinitis; RC: rhinoconjunctivitis; U: urticaria; AB: asthma bronchiale; AP: allergic pharyngitis; SPT: skin prick test; Der p: *Dermatophagoides pteronyssinus*; Der f: *Dermatophagoides farinae*; n.k.: not known; n.d.: not done;
(DOC)

Table S3 **Demographic, clinical and immunological characterization of ten HIV-infected patients with low CD4 counts.** Displayed are age, sex, HIV status, year and CD4 counts when AIDS was diagnosed, CD4 counts at the date of IgE serology and range of CD4 counts for follow-up sera, viral load, antiretroviral therapy, allergic symptoms, year when allergy was diagnosed, positive allergy diagnosis results obtained by skin prick test and IgE serology by CLA assay or Euroline IgE assay. Abbreviations: F: female; M: male; R: rhinitis; RC: rhinoconjunctivitis; U: urticaria; AB: asthma bronchiale; AP: allergic pharyngitis; SPT: skin prick test; Der p: *Dermatophagoides pteronyssinus*; Der f: *Dermatophagoides farinae*; n.k.: not known; n.d.: not done;
(DOC)

Note S1 **Allergen molecules tested with ImmunoCAP ISAC.**
(DOC)

## References

[1] Sibanda EN (2003) Inhalant allergies in Zimbabwe: a common problem. Int Arch Allergy Immunol 130: 2–9. 12576728 10.1159/000068377

[2] Katelaris CH, Lee BW, Potter PC, Maspero JF, Cingi C, et al (2012) Prevalence and diversity of allergic rhinitis in regions of the world beyond Europe and North America. Clin Exp Allergy 42: 186–207. 22092947 10.1111/j.1365-2222.2011.03891.x

[3] Kling S, Zar HJ, Levin ME, Green RJ, Jeena PM, et al (2013) Guideline for the management of acute asthma in children: 2013 update. S Afr Med J 103: 199–207. 23656745 10.7196/samj.6658

[4] Obeng BB, Amoah AS, Larbi IA, Yazdanbakhsh M, van Ree R, et al (2011) Food allergy in Ghanaian schoolchildren: data on sensitization and reported food allergy. Int Arch Allergy Immunol 155: 63–73. 21109750 10.1159/000318704

[5] Mpairwe H, Muhangi L, Ndibazza J, Tumusiime J, Muwanga M, et al (2008) Skin prick test reactivity to common allergens among women in Entebbe, Uganda. Trans R Soc Trop Med Hyg 102: 367–373. 18321545 10.1016/j.trstmh.2008.01.017 PMC2628422

[6] Westritschnig K, Sibanda E, Thomas W, Auer H, Aspock H, et al (2003) Analysis of the sensitization profile towards allergens in central Africa. Clin Exp Allergy 33: 22–27. 12534545 10.1046/j.1365-2222.2003.01540.x

[7] Rujeni N, Nausch N, Bourke CD, Midzi N, Mduluza T, et al (2012) Atopy is inversely related to schistosome infection intensity: a comparative study in Zimbabwean villages with distinct levels of Schistosoma haematobium infection. Int Arch Allergy Immunol 158: 288–298. 22398631 10.1159/000332949 PMC3398828

[8] Sibanda E, Gallerano D, Wollmann E, Valenta R (2012) EFIS-EJI African International Conference on Immunity (AICI). Eur J Immunol 42: 1070–1071. 22539278 10.1002/eji.2012700025

[9] Levy JA (1988) Mysteries of HIV: challenges for therapy and prevention. Nature 333: 519–522. 3287174 10.1038/333519a0

[10] Schneider E, Whitmore S, Glynn KM, Dominguez K, Mitsch A, et al (2008) Revised surveillance case definitions for HIV infection among adults, adolescents, and children aged <18 months and for HIV infection and AIDS among children aged 18 months to <13 years–United States, 2008. MMWR Recomm Rep 57: 1–12. 19052530

[11] Castro KG, Ward JW, Slutsker L, Buehler JW, Jaffe HW, et al (1992) 1993 Revised Classification System for HIV Infection and Expanded Surveillance Case Definition for AIDS Among Adolescents and Adults. MMWR Recomm Rep 4: 1–18. 1361652

[12] Lupinek C, Wollmann E, Baar A, Banerjee S, Breiteneder H, et al (2014) Advances in allergen-microarray technology for diagnosis and monitoring of allergy: The MeDALL allergen-chip. Methods 66: 106–119. 24161540 10.1016/j.ymeth.2013.10.008 PMC4687054

[13] Niederberger V, Ring J, Rakoski J, Jager S, Spitzauer S, et al (2007) Antigens drive memory IgE responses in human allergy via the nasal mucosa. Int Arch Allergy Immunol 142: 133–144. 17057411 10.1159/000096439

[14] Romagnani S (1997) The Th1/Th2 paradigm. Immunol Today 18: 263–266. 9190109 10.1016/s0167-5699(97)80019-9

[15] Lupinek C, Marth K, Niederberger V, Valenta R (2012) Analysis of serum IgE reactivity profiles with microarrayed allergens indicates absence of de novo IgE sensitizations in adults. J Allergy Clin Immunol 130: 1418–1420 e1414. 10.1016/j.jaci.2012.06.028 PMC4578245 22867692

[16] Durham SR, Smurthwaite L, Gould HJ (2000) Local IgE production. Am J Rhinol 14: 305–307. 11068655 10.2500/105065800781329492

[17] Egger C, Horak F, Vrtala S, Valenta R, Niederberger V (2010) Nasal application of rBet v 1 or non-IgE-reactive T-cell epitope-containing rBet v 1 fragments has different effects on systemic allergen-specific antibody responses. J Allergy Clin Immunol 126: 1312–1315 e1314. 10.1016/j.jaci.2010.06.008 20673979

[18] Linhart B, Bigenzahn S, Hartl A, Lupinek C, Thalhamer J, et al (2007) Costimulation blockade inhibits allergic sensitization but does not affect established allergy in a murine model of grass pollen allergy. J Immunol 178: 3924–3931. 17339493 10.4049/jimmunol.178.6.3924 PMC2993922

[19] Zhang Y, Meyer-Hermann M, George LA, Figge MT, Khan M, et al (2013) Germinal center B cells govern their own fate via antibody feedback. J Exp Med 210: 457–464. 23420879 10.1084/jem.20120150 PMC3600904

[20] Suzuki K, Grigorova I, Phan TG, Kelly LM, Cyster JG (2009) Visulazing B cell capture of cognate antigen from follicular dendritic cells. J Exp Med 206, 1485–1493. 10.1084/jem.20090209 PMC2715076 19506051

[21] Mazza DS, Grieco MH, Reddy MM, Meriney D (1995) Serum IgE in patients with human immunodeficiency virus infection. Ann Allergy Asthma Immunol 74: 411–414. 7749972

[22] Goetz DW, Webb EL Jr, Whisman BA, Freeman TM (1997) Aeroallergen-specific IgE changes in individuals with rapid human immunodeficiency virus disease progression. Ann Allergy Asthma Immunol 78: 301–306. 9087157 10.1016/S1081-1206(10)63186-9

[23] Maggi E, Mazzetti M, Ravina A, Annunziato F, de Carli M, et al (1994) Ability of HIV to promote a TH1 to TH0 shift and to replicate preferentially in TH2 and TH0 cells. Science 265: 244–248. 8023142 10.1126/science.8023142

[24] Graziosi C, Pantaleo G, Gantt KR, Fortin JP, Demarest JF, et al (1994) Lack of evidence for the dichotomy of TH1 and TH2 predominance in HIV-infected individuals. Science 265: 248–252. 8023143 10.1126/science.8023143

