# Supplementary material for: Persistence of IgE-Associated Allergy and Allergen-Specific IgE despite CD4+ T Cell Loss in AIDS
Source: PLoS One. 2014 Jun 4;9(6):e97893. doi: 10.1371/journal.pone.0097893 (PMC4045723; doi:10.1371/journal.pone.0097893)
Supplement: Table S1 — Allergen extracts tested. Panel of allergen extracts for diagnosis by skin prick testing and IgE serology (CLA assay, Euroline). Abbreviations: Der p: Dermatophagoides pteronyssinus. (DOC) [file pone.0097893.s001.doc]

**Table S1: Allergen extracts tested**

|  | **SPT** | **MAST CLA** | **EUROLINE IgE assay** |
| --- | --- | --- | --- |
| **1** | Bermuda grass pollen | Cat dander | Cat dander |
| **2** | Maize pollen | Dog dander | Dog dander |
| **3** | 5-grass mixture (Cocksfoot, sweet vernal, rye grass, meadow and timothy) | Horse | Horse |
| **4** | Tree pollen mixture (maple, horse chestnut, plane, false acacia and lime) | Guinea pig | Grass mix |
| **5** | Mould mixture (bakers yeast *Saccharomyces cerevisiae* | Hamster | Birch tree |
| **6** | Feather mixture (duck, goose, chicken) | Rabbit | Mugwort pollen |
| **7** | Grain pollen (maize wheat, barley. Oats) | Mugwort pollen | Birch pollen |
| **8** | Weed pollen | Parietaria pollen | Der p |
| **9** | Tree mixture | Ragweed pollen | Der farinae |
| **10** | Compositaae trees | Olive tree pollen | Cat epithelia |
| **11** | Latex | Birch pollen | Horse epithelia |
| **12** | Cockroach (Blatella germanica) | Juniper | Dog epithelia |
| **13** | Corn moth | Grass mix pollen | Cladosporium |
| **14** | Horsefly | Pine mix | Aspergillus |
| **15** | Cat dander | Hazelnut | Alternaria |
| **16** | Dog dander | Peanut | Hazelnut |
| **17** | Rabbit dander | Walnut | Peanut |
| **18** | Horse hair | Almond | Almond |
| **19** | House dust mites (Der p, Der f.) | Egg | Celery |
| **20** | Wheat | Casein | Egg white |
| **21** | Soy | Potato | Egg yolk |
| **22** | Peanut | Celery | Potato |
| **23** | Duck | Codfish | Celery |
| **24** | Banana | Shrimp | Codfish |
| **25** | Carrot | Apple | Cow Milk |
| **26** | Celery | Wheat flour | Alpha-lactalbumin |
| **27** | Lemon | Sesame | Beta- Lactalbumin |
| **28** | Crab | Soy bean | Casein |
| **29** | Prawns | Peach | BSA |
| **30** | Spinach | Latex | Wheat flour |
| **31** | Strawberry | Penicillium | Rice |
| **32** | Sugar beans | Cladosporium | Soya bean |
| **33** | Melon | Aspergillus | Peanut |
| **34** | Cashew | Alternaria | Havelnut |
| **35** | Egg white, Egg yolk, Whole egg | Cockroach | Carrot |
| **36** | Onion | Der p | Apple |
| **37** | Olive |  | Potato |
| **38** | Orange |  |  |
| **39** | Plum |  |  |
| **40** | Apple |  |  |
| **41** | Potato |  |  |
| **42** | Pork |  |  |
| **43** | Chicken |  |  |
| **44** | Tomato |  |  |
| **45** | Garlic |  |  |
| **46** | Mango |  |  |
| **47** | Kiwi |  |  |
| **48** | Avocado |  |  |
