# Supplementary material for: Persistence of IgE-Associated Allergy and Allergen-Specific IgE despite CD4+ T Cell Loss in AIDS
Source: PLoS One. 2014 Jun 4;9(6):e97893. doi: 10.1371/journal.pone.0097893 (PMC4045723; doi:10.1371/journal.pone.0097893)
Supplement: Table S2 — Demographic, clinical and immunological characterization of eleven HIV-infected allergic patients suffering from AIDS according to the CDC classification. Displayed are age, sex, HIV status, year and CD4 counts when AIDS was diagnosed, CD4 counts at the date of IgE serology and range of CD4 counts for follow-up sera, viral load, antiretroviral therapy, allergic symptoms, year when allergy was diagnosed, positive allergy diagnosis results obtained by skin prick test and IgE serology by CLA assay or Euroline IgE assay. Abbreviations: F: female; M: male; R: rhinitis; RC: rhinoconjunctivitis; U: urticaria; AB: asthma bronchiale; AP: allergic pharyngitis; SPT: skin prick test; Der p: Dermatophagoides pteronyssinus; Der f: Dermatophagoides farinae; n.k.: not known; n.d.: not done; (DOC) [file pone.0097893.s002.doc]

Table S2: Demographic, clinical and immunological characterization of eleven HIV-infected allergic patients suffering from AIDS according to the CDC.

| **Demographics** | | | HIV/AIDS | | | | | | **Allergy** | | | |
| --- | --- | --- | --- | --- | --- | --- | --- | --- | --- | --- | --- | --- |
| **Patient #** | **Age** | **Sex** | HIV Status | AIDS  diagnosed | CD4 counts at diagnosis | **CD4 counts at sampling** | Viral load log10 | ART | Allergy diagnosis | **Symptoms** | Positive SPT | Positive CLA or Euroline IgE assay |
| **1** | 40 | F | positive | 2004 | 30 | 115 | n.d. | Lamivudine,  Nevirapine,  Stavudine, | 12/2004 | RC | Der p | Cat, Dog, Horse, Guinea pig, Hamster, Rabbit, Mugwort, Parietaria, Ragweed, Olive tree, Birch, Juniper, Grass mix, Pine mix, Hazelnut, Peanut, Walnut, Almond, Egg, Casein, Potato, Celery, Codfish, Shrimp, Apple, Wheat flour, Sesame, Soy bean, Peach, Latex, Penicillium, Cladosporium, Aspergillus, Alternaria, Cockroach, Der p |
| **2** | 36 | M | positive | 2006 | 130 | 115 | n.d. | Lamivudine, Nevirapine, Stavudine, | 04/2006 | R | maize pollen, grass pollen mixture, feathers, mould mixture, soy | Rabbit, Mugwort, Parietaria, Ragweed, Olive tree, Birch, Juniper, Grass mix, Pine mix, Hazelnut, Peanut, Walnut, Almond, Egg, Casein, Potato, Celery, Codfish, Shrimp, Apple, Wheat flour, Sesame, Soy bean, Peach, Latex, Penicillium, Cladosporium, Aspergillus, Alternaria, Cockroach |
| **3** | 43 | M | positive | 2004 | 3 | 150 | 381600 | Lamivudine, Nevirapine, Stavudine, | 03/06 | R | cotrimoxazole | Der p |
| **4** | 58 | F | positive | 2004 | 41 | 200 | n.d. | Lamivudine, Nevirapine, Stavudine, | 07/04 | R | Der p | Der p |
| **5** | 37 | F | positive | 2011 | 53 | 77 | 170000 | Zidovudine,  Lamivudine, Nevirapine | 2008 | AB | n.d. | Wheat flour, Rice, Soy bean, Peanut, Haselnut, Carrot, Potato, Apple, Grass mix, Birch tree, Mugwort, Der p, Der f, Dog epithelia |
| **6** | 30 | F | positive | 2004 | 58 | 135 | n.d. | Lamivudine, Nevirapine, Stavudine, | 10/2004 | R | Der p  Mould mix  penicilium, milk. | Cat, Dog, Ragweed, Birch, Juniper, Pine mix, Peanut, Walnut, Almond, Egg, Casein, Potato, Celery, Codfish, Shrimp, Apple, Wheat flour, Soy bean, Peach, Latex, Penicillium, Cladosporium, Aspergillus, Alternaria, Cockroach,  Der p |
| **7** | 40 | F | positive | 2005 | 104 | 142 | 47000 | Stavudine,  Lamivudine,  Nevirapine, | n.k. | R, AB | n.d. |  |
| **8** | 39 | M | positive | 2011 | 36 | 171  (120-217) | 56 | Zidovudine, Lamivudine,  Efavirenz | 2011 | R | n.d. | Der p |
| **9** | 47 | F | positive | 2011 | 57 | 108 | 133000 | Yes | 2011 | AB | 5 Grasses Mix, Horse epithelia | Der p, Der f, Dog epithelia |
| **10** | 67 | F | positive | 2011 | 99 | 117  (117-142) | n.d. | Zidovudine  Lamivudine,  Nevirapine, | 2011 | R | Meat, Carp, Horse epithelia, hens egg yolk, goat epithelia, hen feather, reed, grasses, cereals, Der p, Der f, storage mite | Der p, Der f |
| **11** | 25 | F | Positive | 2011 | 4 | 184  (4-184) | 8800 | Zidovudine  Lamivudine,  Nevirapine, | 2011 | U, Angioedema | Mutton, hens egg yolk, grasses, HDM | n.d. |
