# Supplementary material for: Persistence of IgE-Associated Allergy and Allergen-Specific IgE despite CD4+ T Cell Loss in AIDS
Source: PLoS One. 2014 Jun 4;9(6):e97893. doi: 10.1371/journal.pone.0097893 (PMC4045723; doi:10.1371/journal.pone.0097893)
Supplement: Table S3 — Demographic, clinical and immunological characterization of ten HIV-infected patients with low CD4 counts. Displayed are age, sex, HIV status, year and CD4 counts when AIDS was diagnosed, CD4 counts at the date of IgE serology and range of CD4 counts for follow-up sera, viral load, antiretroviral therapy, allergic symptoms, year when allergy was diagnosed, positive allergy diagnosis results obtained by skin prick test and IgE serology by CLA assay or Euroline IgE assay. Abbreviations: F: female; M: male; R: rhinitis; RC: rhinoconjunctivitis; U: urticaria; AB: asthma bronchiale; AP: allergic pharyngitis; SPT: skin prick test; Der p: Dermatophagoides pteronyssinus; Der f: Dermatophagoides farinae; n.k.: not known; n.d.: not done; (DOC) [file pone.0097893.s003.doc]

Table S3: Demographic, clinical and immunological characterization of ten HIV-infected patients with low CD4 counts.

| **Demographics** | | | HIV/AIDS | | | | | | **Allergy** | | | |
| --- | --- | --- | --- | --- | --- | --- | --- | --- | --- | --- | --- | --- |
| **Patient #** | **Age** | **Sex** | HIV Status | AIDS  diagnosed | CD4 counts at diagnosis | **Range of CD4 counts** | Viral load log10 | ART | Allergy diagnosis | **Symptoms** | Positive SPT | Positive CLA, Euroline IgE assay or allergen sources in ISAC |
| **12** | 49 | M | positive | 2009 | 62 | 168-205 | n.d. | Tenofovir, Emtricitabine, Efavirenz | 2011 | RC | n.d. | Alpha-lactalbumin, Der p, Dog epithelia, Cypress, Cedar, |
| **13** | 32 | F | positive | n.k. | n.k. | 62-106 | n.d. | n.k. | n.k. | R, AP | n.d. | Cedar, Bermuda grass, Cypress, Timothy grass |
| **14** | 50 | F | positive | n.k. | n.k. | 110-677 | n.d. | n.k. | 2011 | AB | Der p, Der f, 4 grains mix | Bermuda grass, Cypress, Der p, Der f |
| **15** | 37 | M | positive | n.k. | n.k. | 375-518 | n.d. | n.k. | 2011 | R | Der p, Der f, cat, peanut, soy, beans | Der p, Der f, Dog epithelia, Bermuda grass, Plane, Wesp, Almond, Peach |
| **16** | 43 | M | positive | 1998 | 461 | 244-288 | 14800 | Zidovudine, Lamivudine,  Efavirenz | n.k. | R, AP | n.d. | Cypress, Cedar |
| **17** | 26 | F | positive | 2009 | 136 | 261-741 | 75930 | Zidovudine, Lamivudine,  Tenofovir | 2010 | R | Glutein, peanut | Altanaria, Cypress, Cedar, Der p, Der f, Walnut, Timothy grass, Bermuda grass, Plane |
| **18** | 39 | F | positive | 2011 | 247 | 209-223 | n.d. | Zidovudine  Lamivudine,  Nevirapine, | 2011 | R | Potato | Der p, Der f |
| **19** | 50 | M | positive | 2001 | 220 | 257-415 | n.d. | Didanosine, Indinavir, Lopinavir, Ritonavir | 2011 | R | Der p, Der f, Meat | Aspergillus, Der p, Der f |
| **20** | 40 | F | positive | 2011 | 488 | 488-722 | <d.l. | Stavudine, Lamivudine, Nevirapine | 2011 | n.k. | n.d. | Der p, Der f, Dog epithelia, Alternaria, Cypress, Bermuda grass, Cedar, Olive, Plane, Timothy grass |
| **21** | 47 | M | positive | 2001 | 156 | 486-501 | <d.l. | Efavirenz, Tenofovir, Abacavir | 2011 | R | n.d. | Cypress, Cedar, Der p, Der f, Latex |
