# Supplementary material for: Persistence of IgE-Associated Allergy and Allergen-Specific IgE despite CD4+ T Cell Loss in AIDS
Source: PLoS One. 2014 Jun 4;9(6):e97893. doi: 10.1371/journal.pone.0097893 (PMC4045723; doi:10.1371/journal.pone.0097893)
Supplement: Note S1 — Allergen molecules tested with ImmunoCAP ISAC. (DOC) [file pone.0097893.s004.doc]

**Supplemental note S1**: Allergen molecules tested with ImmunoCAP ISAC

**Food allergens**: nAct d 1, nAct d 2, nAct d 5, rAct d 8, rAna o 1, rAna o 2, rAna o 2, nAna o 2, rAna o 3, nAra h 1, rAra h 1, rAra h 2, nAra h 3, rAra h 3, nAra h 6, nAra h 6, rAra h 8, rAra h 9, rBer e 1, nBos d 4, nBos d 4, nBos d 5, nBos d 5, nBos d Lactoferrin, nBos d 8, nBos d 8, r aS1-casein, r aS2-casein, r b-casein, rK-casein, nTransferrin, nBos d 6, nBSA, rChe a 1, rCor a 1.0401, rCor a 8, nCor a 9, nFag e 2, rGad c 1, nGal d 1, nGal d 2, nGal d 3, nGal d 5, rGly m 4, nGly m 5, nGly m 6, nJug r 1, nJug r 2, nJug r 3, rMal d 1, nPen m 1, nPen m 2, nPen m 4,rPis v 3, rPru p 1, rPru p 3, rPru du 3, rPru du 4, nPru du 6, rPru du 6.01, rPru du 6.02, nSes i 1, rTri a 14, nTri a 19.0101, nTri a aA_TI, rTri a 36 191, rTri a 36, rTri a 25, rTri a GST, rTri a 32, rTri a 12, rTri a 35, rTri a 37, rTri a 14

**Mite allergens**: rBlo t 5, nDer f 1, rDer f 2, nDer p 1, rDer p 2, rDer p 4, rDer p 5, rDer p 7, rDer p 10, rDer p 11, rDer p 14, rDer p 15, rDer p 18, rDer p 21, rDer p 23, rDer p clone 16, rLep d 2,

**Animal dander allergens**: rCan f 1, rCan f 2, nCan f 3, rCan f 4, rCan f 5, rCan f 5, rCan f 6, rEqu c 1, nEqu c 3, rFel d 1, nFel d 2, rFel d 4, nMus m 1,

**Grass pollen allergens**: nCyn d 1, rPhl p 1, rPhl p 2, nPhl p 4, rPhl p 5, rPhl p 6, rPhl p 7, rPhl p 11, rPhl p 12

**Tree pollen and weed pollen allergens**: rAln g 1, nAmb a 1, nArt v 1, nArt v 3, rBet v 1, rBet v 2, rBet v 4, nCry j 1, nCup a 1, rMer a 1, nOle e 1, nOle e 7, rOle e 9, rPar j 2, rPla a 1, nPla a 2, rPla a 3, rPla l 1, nSal k 1,

**Mould allergens**: rAlt a 1, rAlt a 6, rAsp f 1, rAsp f 3, rAsp f 6, rCla h 8,

**Insect venom allergens**: rPol d 5, rVes v 1, rVes v 5, rVes v 5

**Parasite and insect allergens**: rAni s 1, rAni s 3, rBla g 1, rBla g 2, rBla g 5, nBla g 7,

**Latex allergens**: rHev b 1, rHev b 3, rHev b 5, rHev b 6.01, rHev b 8,

**CCD-Marker**

nMUXF3
